# Environmental exposures and their genetic or environmental contribution to depression and fatigue: a twin study in Sri Lanka

**DOI:** 10.1186/1471-244X-10-13

**Published:** 2010-02-02

**Authors:** Harriet A Ball, Sisira H Siribaddana, Athula Sumathipala, Yulia Kovas, Nick Glozier, Peter McGuffin, Matthew Hotopf

**Affiliations:** 1 MRC Social Genetic and Developmental Psychiatry Centre, Institute of Psychiatry, King's College London, London, UK; 2 Sri Lanka Twin Registry, Institute of Research and Development, Battaramulla, Sri Lanka; 3 Section of Epidemiology, Institute of Psychiatry, Kings College London, London, UK; 4 Goldsmiths, University of London, London, UK; 5 Sydney Medical School, University of Sydney, Sydney, Australia; 6 Department of Psychological Medicine, Institute of Psychiatry, Kings College London, London, UK

## Abstract

**Background:**

There is very little genetically informative research identifying true environmental risks for psychiatric conditions. These may be best explored in regions with diverse environmental exposures. The current study aimed to explore similarities and differences in such risks contributing to depression and fatigue.

**Methods:**

Home interviews assessed depression (lifetime-ever), fatigue and environmental exposures in 4,024 randomly selected twins from a population-based register in the Colombo district of Sri Lanka.

**Results:**

Early school leaving and standard of living showed environmentally-mediated effects on depression, in men. In women, life events were associated with depression partly through genetic pathways (however, the temporal order is consistent with life events being an outcome of depression, as well as the other way around). For fatigue, there were environmentally mediated effects (through early school leaving and life events) and strong suggestions of family-environmental influences.

**Conclusions:**

Compared to previous studies from higher-income countries, novel environmentally-mediated risk factors for depression and fatigue were identified in Sri Lanka. But as seen elsewhere, the association between life events and depression was partially genetically mediated in women. These results have implications for understanding environmental mechanisms around the world.

## Background

Classical twin studies can tell us the degree to which individual differences in a trait or disorder are due to nature or nurture, but they do not tell us which particular environmental exposures are involved. Previously identified socio-environmental risk factors for depression include stressful life events, poor parental care, poverty, low educational qualifications and low social status [1-3]; many of these are also risk factors for fatigue, although the association with social class is less consistent, and fatigue has been associated with overprotective rather than neglectful parenting [4-9]. However, such epidemiological findings can be prone to confounding by genetic effects or the general family environment. The gene-environment correlation, *r*_GE_, is a process in which a person is more likely to be exposed to an environment because of their genetic profile, for example their inherited characteristics might lead them to seek out or evoke certain environmental exposures (see [10] for a review). Twin studies have found that *r*_GE _contributes to the link between negative or stressful life events and depression [11-13], although this was not found in a sib-pair sample that objectively rated life events rather than relying on self or parent reports (which are more likely to be contaminated by depressed mood) [14]. Another twin study examined the link between premorbid stress and chronic fatigue, and found it to be environmentally rather than genetically mediated [15]. Very few other environmental exposures have been examined in this way. Non-Western societies are underrepresented in the psychiatric research literature [16,17], and the higher prevalence of certain environmental exposures compared to Western societies could help to identify risk mechanisms. This study explored potential *r*_GE _between four measured environmental exposures (early school leaving, standard of living, life events, and parental care) and their link with depression and fatigue in Sri Lanka, in order to examine the degree to which environmental effects are free from genetic confounding, and whether such effects are specific to one disorder or a cause of comorbidity.

## Methods

The study received approvals from the Institute of Psychiatry, King's College London Research Ethics Committee; the Ethical Review Committee, University of Sri Jayewardanepura; and the World Health Organisation's Research Ethics Committee.

### Study design and participants

This was a population based twin study, the twin component of the **Co**lombo **T**win **A**nd **S**ingleton **S**tudy (CoTaSS). Full details of the design and implementation of the study are described elsewhere [18]. Briefly, the study took place in the Colombo District of Sri Lanka, an area with population of 2.2 M which includes the island's capital, and varies from urban to semi-urban areas. We added a question to the update of the annual census, asking whether the householder knew of any twins, and identified 19,302 individual twins by this method. Of these, we randomly selected 4,387 individual twins who were at least 15 years old to take part in the project on common mental disorders. Four thousand and twenty four (91.7%) participated, including 1,954 complete twin pairs. We included all consenting individuals aged 15 years or older who spoke sufficient Sinhala to understand the interview. Among men, the mean age was 33 years (s.d. 13); among women the mean age was 35 years (s.d. 14); 46% of the participants were men.

### Data collection

Specially trained research workers visited the subjects' homes to interview them each separately. Interviews and questionnaires were translated. We used the Composite International Diagnostic Interview [19], because it is a structured diagnostic interview for use by lay interviewers, capable of giving DSM/ICD life-time diagnoses for mental disorders. We defined depression according to DSM-IV guidelines except we disregarded the requirement for functional impairment (this was because it was found to be too restrictive in defining depression in this population) [20]. We also did not include opt-outs due to bereavement or mixed states. The current analyses pertain to lifetime-ever history of depression, rather than current depression.

The Chalder Fatigue Questionnaire [21] was administered. 'Abnormal fatigue' was defined as having at least three of the 11 symptoms present at least 'more than usual' over the past month. There were no medical exclusions.

The interview also measured numerous exposures that are potential risk factors for depression or fatigue. Early school leaving and standard of living were examined because they were identified as potential causal factors in an epidemiological analysis of depression in this sample [20]; life events and parental care were added because they have been heavily implicated as risk factors for depression and fatigue respectively [9,22].

Current standard of living was assessed based on a government questionnaire which formed part of the national census. Items tapped into a wide spectrum of household characteristics rather than just detecting the lowest end of the distribution. However, certain items were particularly associated with a history of depression, but only in men [20]. These were: informal structural materials of the abode (*e.g. *metal sheet roof), poor toilet or water facilities (*e.g. *pit latrine toilet, toilet shared with other households or drinking water source shared with other households), and hunger due to poverty in the past three months. The first two were assessed by interviewers' ratings, the last through the subject's self report. These three risk variables correlated with one other 0.37-0.53 within individuals (polychoric correlations). A binary indicator 'standard of living' was created based on a positive score for any of these three environmental risks. We also asked how many months each participant had worked over the past year, because income might account for much of the association between standard of living and history of depression, in the reverse causal direction, particularly in men.

A separate item assessed the length of schooling the subject had received. This was dichotomised to index previously identified risk: those with 10 or fewer years of education were more likely to report a history of depression ('early school leaving') [20].

Life events were assessed using the List of Threatening Experiences (Brief Life Events Questionnaire) [23] over the past 12 months. For the current study we used only those items that could be potentially considered behaviour-dependent, in order to assess the potential of the individual subject to elicit his/her own negative experiences. Also, events that could be 'shared' across both twins in a pair, such as parental death, were not included. Thus the items used were: Separation due to marital difficulties or broken off a steady relationship; serious problem with close friend, neighbour or relative; made redundant or sacked from job; became unemployed or seeking work unsuccessfully for more than one month; major financial crisis; problems with the police involving a court appearance. Each participant received a score indicating having had 0, 1 or 2 or more such experiences over the past year.

The Childhood Experience of Care and Abuse Questionnaire (CECA-Q) was used to assess retrospective self-reports of Neglect (8 items) and Antipathy (8 items) [24] (each of the items were scored on a 5 point scale from "definitely" to "not at all"). These were highly correlated (r > 0.70) and also strongly correlated across reports for mother and father (*r*:0.45-0.58), so these data were combined into one overall variable 'parental care' in order to reduce collinearity and multiple testing. Age and sex mean effects were regressed out separately within same-sex and opposite-sex pairs.

Zygosity was assessed using a validated questionnaire [25,26] administered to both twins.

31.0% of male twins and 25.5% of female twins reported living in the same household as one another. These twins will necessarily share aspects of their 'standard of living' rating (structural materials, and toilet and water facilities of the abode).

A payment of 300 Rupees (approximately £1.50) was offered in compensation for participants' time, at the end of the interview (compensatory payment was not mentioned in the information provided prior to the interview). A substantial percentage of the participants refused the payment and instead requested it to be donated back to the research project [18].

### Analysis

A database was constructed and regression analyses were performed in Stata version 10.1 for Windows. These analyses were corrected for the non-independence of twins within pairs, using the 'cluster' option in Stata. Structural equation model fitting was performed in Mx.

### Phenotypic associations

Odds ratios with depression (history) or fatigue were calculated for each of the four measured exposures (standard of living, early school leaving, life events, and parental care). These were adjusted for age, sex and ethnicity, on the basis that these factors exist prior to illness onset and cannot be an outcome of illness. The odds ratios were also fully adjusted so as to be independent of the other three measured exposures. Moderation by sex was assessed for each association (controlling for age and ethnicity).

### Aetiology of measured exposures

Structural equation models were run to decompose the variance in the measured exposures into that due to genetic (A), shared (family) environmental (C) and unique environmental (E) influences. For the analysis of the continuous variable (parental care), sex effects were tested in the order: i) variance difference; ii) qualitative aetiological difference (whether the same genes and environmental factors are important in both sexes, which is tested by equating genetic and environmental correlations, *r*_A _and *r*_C_, across opposite- and same-sex DZ pairs); iii) quantitative aetiological difference (whether the magnitude of the genetic and environmental influences is constant across sex). Binary variables are assessed assuming a normally distributed latent liability to the exposure, and hence it was not possible to test for sex differences in variance distributions in standard of living, early school leaving and life events, but qualitative and quantitative aetiological sex differences were tested. In addition, a correction parameter to control for age was added to the model for the thresholds for the binary analysis of early school leaving (beta = 0.25, t = 12.44, p < 0.001), because this risk exposure was more common among older participants.

### Aetiology of the overlap between measured exposures and depression or fatigue

Any phenotypic correlation between an exposure and a disorder must at root be due either to genes or environments. The correlation can also be divided into nonfamilial influences that have different impacts on each twin in a pair (E, chiefly found by examining dissimilarities within MZ pairs), or familial influences that make twins similar to one another which includes shared upbringing (C) plus the extent to which they share genetic inheritance (A). Familial influences are assessed by looking for similarity within pairs of twins. Cross-twin logistic regression models, making use of zygosity information, were run to examine the aetiology of the relationship between the measured exposures and depression or abnormal fatigue.

#### Unique environment (E) and potential reverse causation

We first examined the extent to which differences in measured exposures within MZ pairs were associated with differences in phenotype, using an ordered logistic regression model ("MZ differences" model). This would indicate a role for 'E' in the overlap between the exposure and the disorder, in other words whether the exposure is associated with the disorder, unconfounded by genes or shared family upbringing. This suggests a causal association, but it is still possible that the causal direction could run from the disorder to the exposure. Such a reverse causal direction is unlikely to be the case as regards associations with early school leaving assuming post-childhood onset in the majority of cases reporting history of depression, because 95% of the risk group - people reporting 10 or fewer years in education - had left school by age 16, and 100% of these had left school by 21. However, there could still be earlier influences accounting for such associations, such as childhood deprivation. Any 'E' association between lifetime-ever depression and past-year life events is likely to represent a mix of causes and outcomes of depression, but such 'reverse causality' is less likely to be a problem between past-year life events and past-month fatigue. Current standard of living might be an outcome of health status; thus where we found an "MZ differences" association between standard of living and disorder, we tested this further. Finally, although parental care is temporally prior to the assessment of the disorders, current mood could have biased its retrospective reporting.

Note that whilst the 'E' component of the univariate models incorporates the error of measurement in individual variables, this is not the case in the "MZ differences" models that examine the aetiology of the association between the measured exposure and the disorder (unless measurement error is correlated across the exposure and the disorder, or across their reporting).

#### Genetic effects (A) and shared environmental effects (C)

Using both MZ and DZ pairs, we examined to what extent a person's disorder status was associated with their co-twin's exposure, using logistic regression models. This tests whether depression (lifetime-ever) or fatigue is associated with familial susceptibility to the exposure. We next tested whether the familial effect was greater in MZ than in DZ pairs. This would indicate genetic mediation of the familial effect, meaning that the same genes lead to both the exposure and the disorder (*r*_GE_).

If no genetic effect is found, then the familial association between exposure and disorder is likely due to environmental effects of the family of origin (C), through shared upbringing or influence of the family later in life. However, if this is the case, it would not be clear whether the measured exposure is directly involved as the causal component in the family's influence, or if there is a degree of confounding by other environmental factors influenced by the family. Either way, such a result would suggest that there is an overall familial influence (C) on the disorder, a finding that is typically difficult to detect in the classical twin design.

The temporal order of familial associations is unlikely to point to reverse causality: an exposure in one twin is unlikely the result of his co-twin having the disorder. Thus these associations represent some form of familial vulnerability that influences both exposure to an environment and susceptibility to a disorder.

The above models were run separately for men and women when examining history of depression, due to sex differences in the univariate heritability of depression in this population [27]. This was not the case for abnormal fatigue (submitted: [28]) so the models were run combined across men and women as well as separately for each sex.

These logistic regression models do not assume underlying bivariate normality between the exposure and the depressive outcome, as would be the case in structural equation models (SEM) based around polychoric correlations. Prior analyses showed the importance of step-wise relationships between measured exposures and history of depression, rather than an association across the whole continuum of exposures [20]. Thus, these models can be more intuitively interpreted than those based on bivariate normality. Also, focusing on exposure risk categories, and cases versus controls in logistic regression (rather than SEM based on polychoric correlations) allowed sufficient power to examine the associations using narrower definitions of depression (with lower prevalence), and when the associations were only modest [29]. Finally, regression models can be more flexibly used to find out whether A, C or E are involved in an association, whilst controlling for measured potential confounding factors.

## Results

### Descriptive statistics

A history of depression was present in 11.1% of the sample (8.2% of men and 13.6% of women, sex difference: z = 4.98, p < 0.001). Abnormal fatigue was present in 25.3% (21.4% of men and 28.6% of women, sex difference: z = 4.64, p < 0.001). The risk exposures were present in the following proportions: early school leaving: 35.4%; poor standard of living: 20.9%; one life event in past 12 months: 21.3%, 2 or more life events: 8.3%. Parental care was analysed continuously, but 9.5% of participants recorded a score above the previously defined cut-offs [24] indicating either severe antipathy or neglect, by either mother or father. The correlations among the environmental exposures varied from -0.12 to 0.38 (see footnote to Table 1).

**Table 1:** Phenotypic overlap between depression and measured environments (within individuals)

Measured environment	Sex group	OR adjusted for age, sex, ethnicity, plus all the environments	
		
		Depression (lifetime-ever)	Abnormal Fatigue
Early school leaving	Men	0.96 (0.64-1.44)	1.29 (0.96-1.72)
	Women	1.39 (1.01-1.91)	1.45 (1.13-1.87)
	All	1.20 (0.94-1.55)	1.38 (1.14-1.67)

Standard of Living	Men	1.60 (1.07-2.40)	1.58 (1.15-2.16)
	Women	0.83 (0.59-1.18)	1.27 (0.96-1.68)
	All	1.07 (0.81-1.40)	1.39 (1.13-1.72)

Life Events	Men	2.54 (2.02-3.21)	2.29 (1.91-2.74)
	Women	2.62 (2.16-3.18)	1.95 (1.64-2.31)
	All	2.60 (2.24-3.01)	2.10 (1.85-2.38)

Parental care (continuous)	Men	0.88 (0.80-0.96)	0.91 (0.86-0.97)
	Women	0.93 (0.88-0.98)	0.88 (0.85-0.92)
	All	0.92 (0.87-0.96)	0.89 (0.86-0.92)

The correlations among the environmental exposures are as follows:

i) early school leaving with standard of living: 0.38; with life events: 0.18; with parental care: -0.07. ii) standard of living with life events: 0.32; with parental care: -0.03. iii) life events with parental care: -0.12.

### Phenotypic (within-person) associations

The four measured exposures were all independently associated with a history of depression (Table 1), except early school leaving which was marginally non-significant (OR 1.20, 0.94-1.55), and standard of living which only showed a significant association in men. The strength of the association varied by sex only for standard of living (OR 1.60 in men and 0.83 in women, z = 2.52, p = 0.012).

Abnormal fatigue was independently associated with all of the measured exposures, with no significant interactions by sex. Furthermore, all the associations were in the same direction as with depression, i.e. early school leaving, poor standard of living, more stressful life events and neglectful/cold parenting were associated with both fatigue and history of depression.

### Aetiology of measured exposures

The genetic models for early school leaving, standard of living and life events showed a good fit to the data, and the variance components could be equated across sex (Table 2).

**Table 2:** Aetiology of measured environments - univariate ACE models

Measured environment	Sex group	Variance Components			Fit		
					
		A	C	E	Δ χ^2^	Δ df	P
Early school leaving	Male	0.53 (0.15-0.74)	0.36 (0.18-0.71)	0.10 (0.06-0.18)	1.907^1^	1	0.167
	Female	0.35 (0.13-0.65)	0.57 (0.28-0.77)	0.08 (0.04-0.13)			
	**Combined**	**0.45 (0.31-0.60)**	**0.46 (0.32-0.59)**	**0.09 (0.06-0.13)**	**1.766^2^**	**2**	**0.413**

Standard of living	Male	0.16 (0.00-0.44)	0.60 (0.36-0.79)	0.23 (0.15-0.32)	0.398^1^	1	0.528
	Female	0.22 (0.00-0.47)	0.55 (0.34-0.77)	0.22 (0.15-0.32)			
	**Combined**	**0.20 (0.00-0.40)**	**0.57 (0.41-0.73)**	**0.23 (0.17-0.30)**	**0.095^2^**	**2**	**0.953**

Life events	Male	0.34 (0.00-0.59)	0.13 (0.00-0.45)	0.53 (0.41-0.66)	0.441^1^	1	0.507
	Female	0.45 (0.14-0.57)	0.02 (0.00-0.27)	0.53 (0.42-0.65)			
	**Combined**	**0.44 (0.20-0.55)**	**0.03 (0.00-0.22)**	**0.53 (0.45-0.62)**	**0.457^2^**	**2**	**0.796**

Parental care (continuous)	Male	0.36 (0.15-0.59)	0.28 (0.07-0.47)	0.36 (0.31-0.42)	14.020^3^ 4.641^4^	8 1	0.081 0.031
	Female	0.12 (0.003-0.30)	0.45 (0.29-0.57)	0.43 (0.37-0.48)			
	**Combined**	**0.22 (0.09-0.36)**	**0.39 (0.25-0.50)**	**0.40 (0.36-0.44)**	**3.829^2^**	**2**^2^	**0.147**

Best fitting model shown in bold

^1^Fit of ACE model to fully saturated model

^2^Fit of model dropping quantitative sex differences compared to models with A, C and E parameters estimated separately for males and females.

^3^Fit of scalar ACE model to fully saturated model

^4^Fit of model dropping qualitative sex differences

The best fitting model for early school leaving was mainly influenced by A and C factors, with a small contribution from unique environmental influences. Standard of living was heavily environmentally influenced, with only 20% of the variance estimated as due to genetic factors and over a half due to environments shared within the family. The large shared environmental influence is probably partly due to some twins currently living in the same household, slightly more so among men than women, which could also account for the larger effect of shared environments in males. However, the total shared environmental influence (60% in men and 48% in women) cannot be entirely accounted for by this, because under a third of twin pairs lived together. In the model for life events, the A and E factors each influenced roughly half of the variance.

A scalar model was used for parental care (because of sex differences in variance: 5.4 in men, 7.9 in women, p < 0.01). The fit was poor (18.661, df = 9, p = 0.028) until the shared environmental correlation between males and females within opposite sex DZ pairs was allowed to be less than unity (Δ χ^2 ^= 14.020 for 8 df, p = 0.081). Accordingly, the fit worsened when *r*_C _and *r*_A _were fixed at 1.0 and 0.5 respectively (4.641, 1 df, p = 0.031), indicating that there are qualitatively different environmental (or genetic) factors influencing parental care as reported by men and women. However, the *magnitude *of the influence of A, C and E did not differ across sex (3.829 for 2 df, p = 0.147). The best fit model had a moderate genetic contribution and larger contributions from C and E.

These results suggest that the measured exposures were mostly environmental in origin (rather than being mostly expressions of genetic tendencies). Shared family environments were particularly important for early school leaving, standard of living and parental care.

### Aetiology of the association between depression/fatigue and measured exposures

#### E: unique environmental associations (not confounded by genes or family upbringing)

The "MZ differences" regression models revealed that, among men, three of the measured exposures (standard of living, early school leaving, and life events) were associated with history of depression through the influence of nonshared environments (E) (Table 3, and for a summary of results see Table 4). Furthermore, these 'E' influences in men were significant independent of one another (ordered logistic regression model simultaneously including all three exposures gave OR for early school leaving 4.02, 95% CIs 1.73-9.38; standard of living 2.43, 1.07-5.51; life events 1.59, 1.03-2.48). Early school leaving is likely to be temporally prior to depression onset, but the association with life events may well be at least partly an outcome of depression. To control for the possibility that depression might have driven the association with standard of living (in men) through reduction in work capacity, we ran a further model that adjusted for the MZ differences in amount of work done over the past year. The E association still remained independent of any association with work (2.41, 1.07-5.43). This finding reduces the likelihood of one pathway of reverse causation, but there still could be others, or the effect on work could have been longer ago than the previous year. In women, there were no associations with history of depression mediated by 'E'.

**Table 3:** Aetiology of the association between depression and measured environments

Measured environment	Sex group	Time period	Depression (lifetime-ever)		
			
			MZ differences OR (95% CI) 'E'	Interaction: familiality^1^ X zygosity, z score (p) 'A'	Familiality^1^ OR (95% CI)
Early school leaving	Men	Prior to age 16 in 95% of cases	4.12 (1.81 - 9.41)	0.82 (0.413)	0.66 (0.40-1.10)
	Women		1.68 (0.77-3.63)	0.70 (0.484)	1.34 (0.94-1.90)

Standard of Living	Men	Current	2.37 (1.06 - 5.31)	0.93 (0.350)	1.00 (0.59-1.72)
	Women		0.67 (0.34-1.30)	1.09 (0.277)	1.19 (0.82-1.74)

Life Events	Men	Past year	1.98 (1.29-3.03)	0.15 (0.877)	1.50 (1.11-2.03)
	Women		1.27 (0.89-1.83)	1.99 (0.046)^2^	1.61 (1.27-2.04)

Parental care (continuous)	Men	Prior to age 17 (retrospective)	1.04 (0.88-1.23)	3.11 (0.002)^3^	0.93 (0.84-1.03)
	Women		1.00 (0.90-1.11)	0.10 (0.920)	0.88 (0.82-0.93)

Logistic regressions examining MZ differences ('E') (predicting within-pair difference in depression from within-pair differences in environments), and familiality ('A' and 'C') (predicting depression from co-twin's environmental experiences, in both MZ and DZ pairs)

^1^Familiality: all twins except DZOS

^2^The cross-twin relationship between life events and depression in women by zygosity was OR 1.97 (1.48-2.63) in MZ pairs, and 1.17 (0.76-1.82) in DZ pairs.

^3^The cross-twin relationship between care and depression in men by zygosity was OR 0.81 (0.73-0.90) in MZ pairs and 1.10 (0.93-1.31) in DZ pairs.

**Table 4:** Summary of findings

Sex	Risk	Depression: mediation by			Fatigue: mediation by		
			
		A	C	E	A	C	E
Men	Early school leaving			+			+
	Standard of living			+		+	
	Life events		+	+*		+	+
	Parental care	+				+	

Women	Early school leaving					+	
	Standard of living					+	
	Life events	+				+	+
	Parental care		+			+	

A: genetics. C: family environments (or family-wide confounds). E: unique environments (i.e., those specific to each person within a twin pair).

*Temporal direction may run from depression to life events

Note that the "MZ differences" (E) association between early school leaving and history of depression is present despite there not being a phenotypic association between the two (Table 1). This does not invalidate the E association but suggests that other, familial, influences are also operating in the opposite direction.

Both early school leaving and life events were associated with abnormal fatigue as nonshared environmental effects (OR 1.98, 95% CI 1.25-3.13, and 1.74, 95% CI 1.41-2.14 respectively, in men and women combined, Table 5, and for a summary of results see Table 4), although the former was not significant in women when examined separately by sex.

**Table 5:** Aetiology of the association between depression and measured environments

Measured environment	Sex group	Time period	Abnormal fatigue (past month)		
			
			MZ differences OR (95% CI) 'E'	Interaction: familiality^1^ X zygosity, z score (p) 'A'	Familiality^1^ OR (95% CI)
Early school leaving	Men	Prior to age 16 in 95% of cases	3.52 (1.79-6.93)	1.31 (0.189)	1.14 (0.81-1.60)
	Women		1.28 (0.68-2.39)	0.54 (0.586)	1.54 (1.18-2.00)
	All		1.98 (1.25-3.13)	1.22 (0.223)	1.37 (1.11-1.68)

Standard of Living	Men	Current	1.46 (0.77-2.76)	0.41 (0.682)	1.71 (1.21-2.40)
	Women		1.07 (0.65-1.77)	1.15 (0.248)	1.79 (1.36-2.36)
	All		1.19 (0.80-1.77)	0.65 (0.516)	1.77 (1.43-2.20)

Life Events	Men	Past year	1.46 (1.05-2.02)	1.07 (0.286)	1.58 (1.29-1.93)
	Women		1.97 (1.49-2.61)	0.40 (0.690)	1.39 (1.16-1.67)
	All		1.74 (1.41-2.14)	0.44 (0.663)	1.47 (1.28-1.68)

Parental care (continuous)	Men	Prior to age 17 (retrospective)	1.00 (0.88-1.13)	0.17 (0.861)	1.58 (1.29-1.93)
	Women		0.94 (0.87-1.02)	0.76 (0.446)	0.91 (0.87-0.95)

Logistic regressions examining MZ differences ('E') (predicting within-pair difference in fatigue from within-pair differences in environments), and familiality ('A' and 'C') (predicting fatigue from co-twin's environmental experiences, in both MZ and DZ pairs)

^1^Familiality: all twins except DZOS

#### A: Genetic mediation

Genetic mediation (i.e. a larger familial association in MZ than DZ pairs) was found for the association between history of depression and life events in women (MZ OR 1.97, 95% CI 1.48-2.63; DZ OR 1.17, 95% CI 0.76-1.82; z = 1.99, p = 0.046) (Table 3). This was also the case for parental care in men (MZ OR 0.81, 95% CI 0.73-0.90; DZ OR 1.10, 95% CI 0.93-1.31; z = 3.11, p < 0.001). These effects suggest that people's genetically-mediated characteristics, for example personality, may elicit aversive exposures from their surroundings, which then predispose them to depression.

There was no evidence of genetic mediation of the familial associations with abnormal fatigue (the associations were not significantly greater in MZs than DZs) (Table 5). This indicates that any familial associations are likely to be due to shared environmental effects.

#### Familial association with no evidence of genetic effects

Familial influences were tested by examining the cross-twin associations between one person's depression (or fatigue) and measured exposure in the co-twin, in both MZ and DZ pairs. This assesses whether the risk for depression or fatigue is greater in people who are familially susceptible to the exposure (i.e., those whose co-twin reported the exposure); shared environments (C) are implicated in the absence of evidence of genetic mediation (A). This revealed familial associations of life events with history of depression for men (1.50, 95% CI 1.11-2.03), and parental care with history of depression for women (OR 0.88, 95% CI 0.82-0.93).

For Abnormal Fatigue, there were significant familial associations with each of the measured exposures: early school leaving (OR 1.37, 95% CI 1.11-1.68), standard of living risk (OR 1.77, 95% CI 1.43-2.20), life events (OR 1.47, 95% CI 1.28-1.68) (all assessed in men and women combined; although that for early school leaving was not significant when examined separately in men), and parental care in men (0.89, 95% CI 0.82-0.96) and women (0.91, 95% CI 0.87-0.95) (parental care was examined separately by sex due to the sex differences described in Table 2). However, these associations could be due to confounding by other familial exposures. Thus the strongest candidates as true environmental contributions to disorder are those identified as having 'E' overlaps with the disorders; 'C' associations identified here still require further investigation in order to support their status as causal risk processes.

## Discussion

This study examined the environmentally-mediated impacts of four notable risk factors for depression and fatigue, in Sri Lanka, where some of these risks are especially prevalent. Exposure to early school leaving, poor standard of living (informal structural materials, poor toilet or water facilities, or hunger due to poverty in the past 3 months), stressful life events and poor parental care in childhood were mainly associated with depression (lifetime-ever) and fatigue through environmental mechanisms, although genetic factors also played a role.

For history of depression, we found person-specific environmental effects "uncontaminated" by gene-environment covariation or family-wide exposures, from early school leaving and standard of living, but only in men. In women, these environmental pathways to depression were not found, but the association between life events and depression was partly mediated by genetics. These measured environments partly explain some of the overall aetiological sex difference in depression, which was found to be less heritable in men in this population [27]. However, we also found a role for genes in depression in men (mediating the link between parenting experiences recalled from childhood and depression). And the association with this same exposure revealed a role for family-wide environmental influences in depression in women. Although these reports might be linked to recall bias rather than experiences in childhood, our findings suggest that there is a genetic component to male depression in this population (which was too small for us to have power to confirm in the previous univariate study) [27]. It also suggests that there are shared (family) environmental influences on depression (although we cannot be sure of the particular aspect of the environment that is responsible).

For fatigue, we found person-specific environmentally-mediated effects from negative life events (which is consistent with twin findings from Sweden [15]) and from early school leaving, but not from standard of living or parenting. In addition, there was a role for family-environmental effects on the relationship between all four risk factors and fatigue.

### Some specificity of environmental influences on depression and fatigue

There were some similarities in that the exposures that influenced fatigue and history of depression, in particular early school leaving (in men) had environmentally mediated effects on both disorders. Thus early school leaving is a strong candidate as an environmentally-mediated risk factor that leads to both depression and fatigue in men. So although the duration of one's school career was found to be partly heritable (this might operate via intelligence which is itself highly heritable [30]), the environmental rather than the genetic influences on school duration are connected to depression and fatigue in later life. In contrast, men's standard of living appears to have an environmental impact on depression but not fatigue, despite both these outcomes often occurring in the same individual, and despite the likelihood of (tiring) manual labour among those with poor standards of living.

The results also highlight the contribution of shared (family-wide) environmental factors (C) to fatigue, and to a lesser extent to history of depression, in Sri Lanka. But it is not clear whether the specific measured risk factors measured here are responsible, or whether other aspects of the family environment that could be acting as confounders. Whilst 'C' has generally not been found to be an important determinant in previous (Western) twin studies of depression or fatigue, it is hard to definitively rule out [31-33]. Thus the present findings might be representing effects specific to Sri Lanka, or they may reflect small 'C' effects that exist throughout the world that have not been confidently detected elsewhere due to low power to detect 'C' in the classical twin design. This highlights the particular importance of controlling for potential confounders within the family when examining risk factors for fatigue.

### Genetic mediation of apparent environmental risks

Where we found genetic mediation ('A') of the association between exposure and disorder, an active or evocative gene-environment correlation (*r*_GE_) is indicated. This means certain characteristics that are partially heritable (e.g. risk taking and other aspects of personality and lifestyle) lead people to seek out or elicit certain environments, which are then associated with the disorder. This was found in relation to life events and history of depression in women, as has been found elsewhere [11,12], and supports findings that this type of association is more characteristic of women than men [13]. There was a lack of genetically-mediated associations of measured exposures with fatigue, despite apparent heritability of this phenotype both in this population (submitted: [28]) and elsewhere [31,34-36]. This suggests that genetic factors are more likely to have a direct impact on fatigue, rather than an indirect effect through influencing personality and/or lifestyle. For example, the genetic factors influencing fatigue might directly influence sensory perceptions, which has been shown to be heritable.

### Limitations

This study is based on cross-sectional reports, and requires confirmation through longitudinal waves of data. Although the findings are based on correlations, the twin structure of the data does mean we can be confident of ruling out genetic and family-environmental confounds by examining differences within MZ pairs. Nonetheless, this is not an interventional study and thus we cannot definitively pinpoint precise events that eventually resulted in depression or fatigue outcome. For example early school leaving might be a marker of earlier environmental effects such as a bad accident that prevented school attendance in one MZ cotwin but not the other. Also, the environmental exposures correlated with one another to some degree; but rather than appearing to be a generalised effect of poverty, we found evidence of independent environmentally-mediated associations of early school leaving, standard of living and life events with depression in men.

The lifetime-ever status of the depression assessment makes it hard to rule out reverse causality for environmentally-mediated associations because the exposure could be relatively recent. So although an environmentally-mediated association of life events with history of depression was detected in men, it is likely that at least some of this association is driven by prior depression.

Current mood or personality may have affected the retrospective reporting of parental care and recent life events. This dictates caution in interpreting within-person associations of these exposures with depression and fatigue.

Finally, although our analyses examining the overlap between measured exposures and fatigue or depression outcome looked for correlations between genes and environments, our assessment of the heritability of depression and fatigue did not assess potential interactions between genes and environments, due to low power. Studies on other samples have found evidence for such interactions in the aetiology of depressive symptoms [37,38].

## Conclusions

This study has identified some specific measured exposures that have non-genetic influences on depression, and some that influence fatigue. It is likely that the extent and magnitude of the effects of standard of living and early school leaving examined here would be too rare to examine in population-based genetically sensitive designs in more developed countries. Thus these novel findings are possible partly because of the unique setting of this large twin study. However, these mechanisms are also likely to operate in other countries where these exposures are less common or less severe.

This study highlights the usefulness of the twin design for understanding environmental as well as genetic mechanisms. It suggests reducing early school leaving could be an important intervention to potentially reduce depression and fatigue outcomes, particularly for men (but further investigations would be required to fully understand these associations). Further exploration of childhood factors may also help elucidate mechanistic pathways leading to chronic fatigue syndrome (such as childhood longstanding illness, shown to be a prospective risk in a UK cohort [6]). The findings also emphasise the need to control for potential confounding mechanisms when examining associations between exposures and outcomes, particularly the role of genetic mediation in depression, and family-wide confounds in fatigue.

## Competing interests

The authors declare that they have no competing interests.

## Authors' contributions

HB undertook the statistical analyses and wrote the first draft. MH and AS were principal investigators, responsible for study's design and implementation. MH and PMcG supervised the statistical analyses and their interpretation. MH, PM, AS & SS designed the study. SS and AS were responsible for over-seeing data collection. NG was responsible for questionnaire design and training. YK contributed to data analysis and its interpretation. All authors contributed to and have approved the final manuscript.

## Pre-publication history

The pre-publication history for this paper can be accessed here:

http://www.biomedcentral.com/1471-244X/10/13/prepub
